# Atomic oxygen photometric temperature of lightning and its sub-processes with SOPAPILLA

**DOI:** 10.1038/s41598-025-34189-8

**Published:** 2026-01-09

**Authors:** Jacob Wemhoner, Adonis F. R. Leal, Caitano L. da Silva, Richard Sonnenfeld

**Affiliations:** Department of Physics & Langmuir Laboratory, New Mexico Tech, Socorro, 87801 USA

**Keywords:** Climate sciences, Physics

## Abstract

Information on the temperature of lightning sub-processes is crucial to constrain the chemical and energetic impacts of lightning. However, the peer-reviewed literature on even the most energetic of these processes is scant. In this article, we present a method for measuring these temperatures using the Spectrally-resolved OPtical Automated Photometric Instrument at Langmuir LAb (SOPAPILLA), a near-infrared photometer array that enables a larger number of temperature measurements than previously possible. Across 23 lightning return strokes in the field of view of the instrument, the average peak temperature was 34.8 kK which was weakly correlated with the peak current as reported by a lightning location network. Additionally, in 33 subsequent return strokes reusing the same channel, the temperature of the precursor dart leader (15 kK) was only 3.5 kK cooler (on average) than the ensuing return stroke. We also recorded a close-range powerful stepped leader that displays stepwise temperature enhancements. Finally, by measuring the temperature of M-components (23.5 kK on average) we determine that a return stroke followed by continuing current managed to sustain a temperature of 20 kK for at least 10 ms, cooling slowly at a rate of 1.5 kK/ms.

## Introduction

In order to understand the fundamental physics of lightning and its impacts on the environment, it is crucial to measure the plasma properties of the lightning channel, such as its temperature, electrical conductivity, energy deposition rate, etc. In this work we shall focus on the temperature. The plasma temperature of the lightning channel governs its nonlinear resistance^1^, which in its turn directly affects lightning phenomenology (e.g., stroke multiplicity^2^). Measuring temperature in a time-dependent manner, and using it to constrain energy deposition, could be pivotal for understanding which lightning return strokes could cause wildfires^3^. Additionally, the temperature drives nitrogen oxide fixation via the Zel’dovich mechanism^4^. Other papers have tried to estimate the impacts of lightning, regarding both its chemical yield^5^ and its potential to start wildfires^6^, by extrapolating conclusions about temperature from measurements of the return stroke peak current. Unfortunately, the lightning temperature and the peak current in the channel seem to be only weakly connected by theory and they make for poor substitutes for one another^7^. So, temperature is a crucial quantity which needs to be abundantly measured in order to advance knowledge in the field^8^.

Modern temperature measurements are based on the slitless spectroscopy technique^9–11^. On the one hand, this method is the most accurate approach available to date. On the other, the method has several main issues, including: large amounts of instrument dead-time between triggers, high sensitivity to the relative spatial deviation between the lightning and the camera, and a small field-of-view (FOV)^7^. As a consequence of these factors, the vast majority of the published works only report the spectra of return strokes^9–20^, with comparatively very little information available in the peer-reviewed literature regarding the spectra of stepped leaders^21,22^, dart leaders^23,24^, and M-components^25,26^. The need for widespread temperature measurements in combination with the limitations in the spectroscopic method led us to look into a more efficient and cost effective method for capturing the temperature of lightning and its sub-processes.

Our search for an instrument that could measure the totality of a lightning strike ended with a photodiode array. We first deployed this array in the Summer of 2023 and reported preliminary results in a previous publication^7^. While photodiodes have been used for lightning observations in the past^27–32^, they were mostly used for power and energy estimates. However, by adding narrow-band filters in front of the photodiodes, we can produce a measurement that is similar to the integrated luminosity over a spectral line in spectroscopy. This allows us to estimate the temperatures of lightning phenomena in greater abundance than the spectroscopic method.

In this work, we report on multi-band observations of lightning, which include data in the near-infrared, optical, and radio bands collected with the Spectrally-resolved OPtical Automated Photometric Instrument at Langmuir LAb (SOPAPILLA). The multi-band view provided by this instrument allows for the detailed characterization of lightning and its sub-processes. We report temperatures and temperature trends for dart leaders, M-components, and stepped leaders sampled at 800 kHz, faster than any previous measurements for these events. Additionally, we describe instrumental improvements that have increased accuracy of the measured photometric temperatures, which include direct measurements of the continuum radiation intensity in the near-infrared.

## Results

SOPAPILLA contains 6 photodiodes in an array. In this article we focus on only 4 of them: three with a 1-nm, narrow-band filter around atomic oxygen emission lines (777, 844, and 926 nm), and a fourth with a wider filter in a spectral region with no transition lines (812±5 nm). This fourth photodiode was added in the Summer of 2024 and it significantly reduces the error bounds on the temperature estimates by measuring the continuum radiation^33^ at a wavelength close to all 3 oxygen lines of interest. In addition to this continuum measurement, the gain on the photodiodes was increased and this allowed us to measure the temperature of a few dimmer lightning sub-processes.

Aside from photodiodes, SOPAPILLA has both a fast and slow antenna with time constants of 500 $\upmu \mathrm s$ and 10 s, respectively. SOPAPILLA automatically triggers based on changes in the electric field measured by the fast antenna. It then sends this trigger to a Chronos camera running at 1,000 frames per second with a 500 $\upmu \mathrm s$ exposure time to add spatial context. Twenty seven lightning flashes were simultaneously recorded with all sensors in SOPAPILLA during the Summer of 2024 (photometers + video + radio), allowing us to easily determine if any part of a flash is in the FOV, and to identify its sub-processes.

### Measurement of continuum radiation improves the accuracy of temperature estimates

**Fig. 1:**
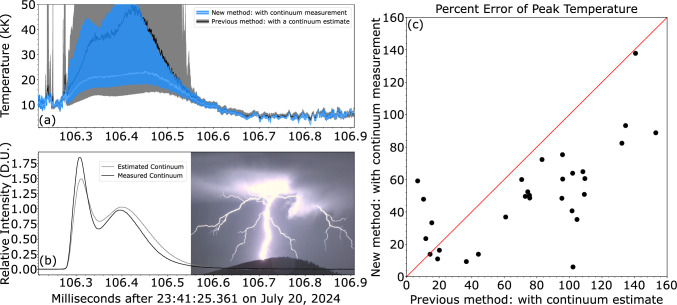
Comparison between temperature and associated uncertainty using a continuum *measurement* and a continuum *estimate*, which assumes that the continuum intensity is 30±20% of the 926 nm intensity^7^. Error weighted temperature with error bars (**a**) and continuum radiation intensity (**b**) as a function of time for a sample return stroke. The inset in panel **b** shows a snapshot of the RS captured with the Chronos camera. **c** Error in peak temperature estimate (in %) defined as the disagreement between the two line ratios. Panel **c** is a generalized version of panel **a** as it shows a comparison for all 30 data points for which both methods yielded a meaningful temperature.

The continuum channel in the photometric array allows SOPAPILLA to measure the intensity of near-infrared radiation in the 807–817 nm band. The continuum measurement in its turn enables for a more constrained estimate of the temperature. The continuum intensity needs to be subtracted from the measured intensity to isolate the contribution of the oxygen line emission, as described in the Methods section at the end of this article. In this section we present comparisons between temperature estimates which use a continuum *estimate* as done in our previous work^7^, with ones using a continuum *measurement*. An example of this is shown in Fig. 1a, b, where a return stroke (RS) happens along a new ground connection in a flash (a snapshot of the RS is shown in Fig. 1b). In our previous work^7^, the temperature was given by estimating that the continuum intensity was 30% of the intensity measured at 926 nm, while the error bars were given by assuming a maximal possible range between 10 and 50%. This wide range of possible continuum estimates dictated the size of the error bars. Meanwhile, with a continuum measurement the temperature error bounds are solely determined by the disagreement between the temperature given from the two different line ratios, 926/777 and 926/844. Figure 1a shows a substantial reduction in the error bar size made possible by the inclusion of a continuum measurement.

To address the validity of the continuum estimate used previously, Fig. 1b compares the continuum estimate as 30% of the 926 nm signal^7^ to the continuum measurement, after a blackbody adjustment for wavelength (see Methods section). It turns out that the estimate is reasonable but not perfect. While the magnitudes are comparable, we can see that the temporal evolution of the two curves has several distinctions. However, to make a fair comparison between the continuum estimate and continuum measurement method we will assume that 30% is accurate enough to use as a direct replacement for the continuum measurement. This allows us to compare the difference in error bars assuming they are given solely by the disagreement between the 2 different ratios for both methods. Using these assumptions for all 95 RSs in our data set, the continuum estimate (as 30% of the 926 nm intensity) failed to yield a meaningful temperature in 65 of the 95 cases due to it being more sensitive to signal-to-noise changes. Figure 1c shows a comparison of error bar size at peak temperature (as % of peak value) for the 30 RSs where both methods worked. The error bars in Fig. 1c and in the remainder of the article are calculated solely from the disagreement between the two line ratios. The use of a continuum measurement reduced the error bars in 87% of the cases (26 cases). Additionally, nearly half of the peak temperatures obtained with the continuum estimate have errors above 100%. The analysis reported here shows that in the absence of a continuum measurement, approximately 27% of the intensity at 926 nm arises from continuum radiation. This figure is very close to the 30% value adopted previously^7^, which was inferred from slitless spectroscopy^11^.

### Temperature of return strokes

**Fig. 2:**
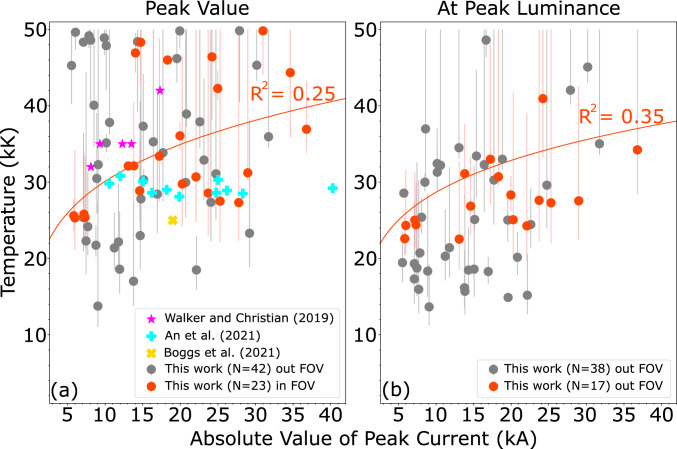
Temperature versus peak current for return strokes with overlapping ENTLN detection. **a** Peak temperature value. **b** Temperature at the instant of peak 777 nm luminance. Vertical lines represent the error in the temperatures obtained from the disagreement between the two different ratios 926/777 and 926/844. The red traces mark power-law fits to the data in the FOV. The R$^2$ value is also displayed.

Figure 2 shows the temperature of all cloud-to-ground (CG) RSs with less than 100% error as a function of peak current reported by ENTLN at 2 different times. Figure 2a reports the peak temperature value and displays a comparison with spectroscopy data available in the literature. Meanwhile, Fig. 2b reports the temperature at the peak luminance of the 777 nm line. In both panels red and gray circles display RSs that are in or out of the FOV, respectively. The average peak temperature for the 23 return strokes in the FOV is 34.8 ±8.3 kK, which is similar to both previous theoretical estimates^1,29^ and previous measurements^9–11^.

Both panels in Fig. 2 show fits in the format *T* = $KI_p^{\alpha }$ for all data in the FOV, where *T* is temperature, *K* and $\alpha$ are fit constants, and $I_p$ is peak current. For the peak temperatures in Fig. 2a, we found that $\alpha$ = 0.21; this exponent is close to the 0.17 figure reported previously^7^, based on the simulations of Taylor et al.^1,34^. Despite a low $R^2$ value, this agrees with the conclusions drawn from an earlier prototype of this instrument^7^ and the general trend from the 4 flashes with spectroscopic temperatures^9–11^, which is that peak temperature naturally has a weak dependence on peak current.

Even with the continuum measurement, the error bars are still large, so we plot the temperature during peak luminance of the lightning return stroke in Fig. 2b as this moment has a better signal-to-noise ratio and the lower temperatures have necessarily lower error bounds. The fit line on Fig. 2b yields $\alpha$ = 0.20. The value is nearly identical to what was found in panel (a), but with a higher $R^2$ value. Fit lines were calculated for the data outside of the FOV but not shown due to the exceptionally-low $R^2$ values. For RSs outside of the FOV in Fig. 2 the $R^2$ value for peak temperatures was 1.1$\times$10$^{-5}$, while the $R^2$ value at peak luminance was 3.4$\times$10$^{-3}$. These incredibly low values indicate that temperatures reported outside of the FOV contain no information about the peak current, while the ones in the FOV do. Additionally, the errors on the temperature tend to be higher if the channel falls outside of the FOV.

### Temperature of other lightning sub-processes

**Fig. 3:**
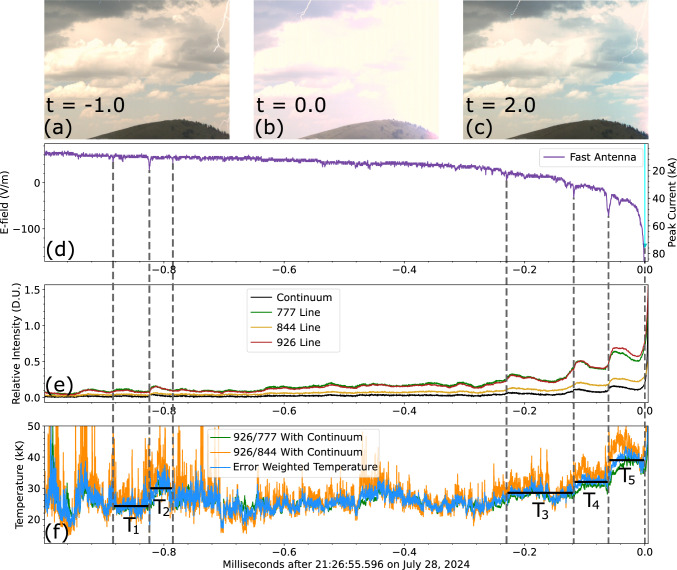
SOPAPILLA recording of a stepped leader 2.7 km away as a function of time. The top row **a**–**c** shows Chronos context images with time shown in ms. **d** Fast antenna field change (left y-axis) and ENTLN peak current (inverted right y-axis). **e** Relative intensity (in digitizer units) measured across the four SOPAPILLA photodiodes. **f** Error-weighted average temperature reported alongside the temperature measurements from the two ratios, there is significant overlap of temperature measurements which leads to some parts of the 926/777 estimate being obscured. The vertical dashed lines mark large leader steps. The black, horizontal traces represent average temperatures across 5 selected intervals before around the large steps (see text for details). The RS data at *t* = 0 and later is saturated due to proximity to sensor and strike intensity.

**Fig. 4:**
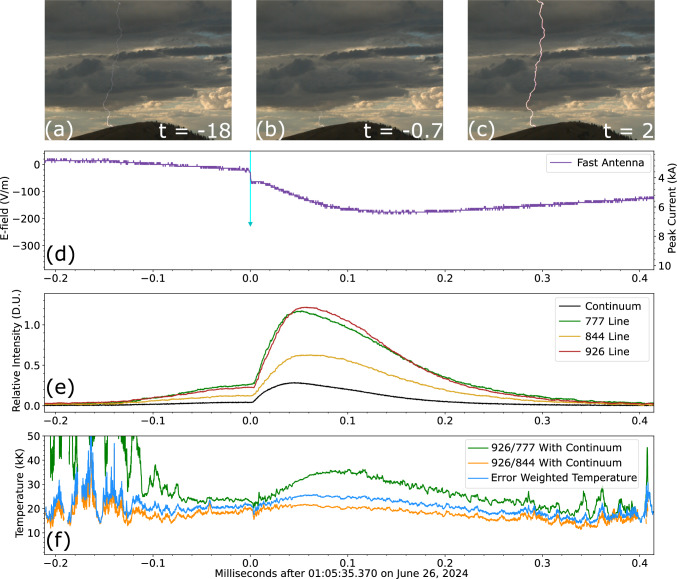
SOPAPILLA recording of a dart leader 4.7 km away. The figure has the same format as preceding Figure 3. Top row **a**–**c** shows Chronos context shots at −18, −0.7, and 2 ms. **d** Fast antenna field change and ENTLN peak current. **e** Photometer array signals. **f** Temperature and error range (from the disagreement between the two line ratios).

**Fig. 5:**
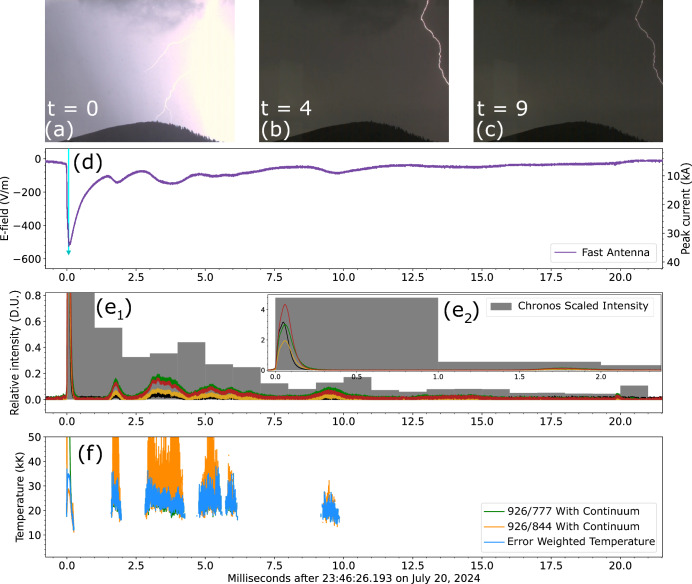
SOPAPILLA recording of a RS, 4.2 km away from Langmuir Lab, which was followed by continuing current superimposed with M-components. The figure has the same format as preceding Figs. 3 and 4. Top row **a**–**c** shows Chronos context shots at 0, 4, and 9 ms. **d** Fast electric field change and ENTLN peak current. **e** Relative intensity from photodiodes (4 curves) and from integrated Chronos camera luminosity (gray bars). Panel **e** is broken down into two intervals: 21-ms record (**e**$\bf _1$) and first 2.4 ms (**e**$\bf _2$). **f** Temperature and error range.

With the additional sensitivity offered by this reconfigured instrument it is often possible to measure the temperature of additional lightning sub-processes beyond the RS. Information on the temperature of leaders and M-components acquired via spectroscopy is sparse in the literature^24,26^. In the remainder of this article we shall cover three types of sub-processes: stepped leaders (Fig. 3), dart leaders (Fig. 4), and M-components (Fig. 5). Stepped leaders are the mechanism by which a lightning flash carves its way to the ground, the leader channels step in a stochastic manner injecting a current surge in the channel. Dart leaders are similar to stepped leaders but they retrace a decayed channel to create a subsequent return stroke. M-components are re-illuminations of a faintly lit channel maintained by continuing current flowing to the ground^35^. RSs with long-lasting continuing currents often have M-components superposed in it^36^. Long continuing current flashes are more likely to start wildfires due to their exacerbated energy deposition^37^.

Figures 3, 4, and 5 all follow a similar layout. They start with 3 frames from the context camera labeled (a), (b), and (c). The fast antenna field change (left side axis) and ENTLN peak current data (right axis) are shown in panel (d). Panel (e) displays light curves from the relevant channels of the photometer array, these light curves are range- and spectral width-normalized so they can be in the same scale. Meanwhile, panel (f) reports the temperature data inferred from both ratios, as well as the error-weighted average temperature. Each figure reports time in milliseconds relative to the horizontal axis label of panel (f).

#### Stepped leader

Figure 3 shows a lightning stepped leader which happened 2.7 km away from the observation site. The stepped leader has an average temperature of 25 kK. This temperature is quite larger than both what was reported by Chang et al.^24^, which was $\sim$15 kK, and the initial modeling attempts for stepped leaders summarized by Rakov and Uman^38^. This number is closer to what was estimated by Orville corresponding to the leader tip^39^. Since the ensuing return stroke peak current was 74 kA, it is plausible that this event may be a particularly powerful stepped leader. While the 25 kK figure may not be representative of all stepped leaders, the observed temperature features remain interesting. The vertical dashed lines across the three panels mark a series of “large steps”. They are characterized by brief surges in the electric field change (Fig. 3d) and in near-infrared luminosity across the four SOPAPILLA channels (Fig. 3e). There is some overlap between the temperature estimates in Fig. 3f but it is still clear that each large step generates an increase in the photometric temperature, marked with black horizontal lines. The average temperatures marked in the figure are: $T_1$ = 24.7 kK, $T_2$ = 29.9 kK, $T_3$ = 28.5 kK, $T_4$ = 32.2 kK, and $T_5$ = 39.0 kK.

#### Dart leaders

The data set contains 33 examples of subsequent return strokes preceded by visible dart leader luminosity, like shown in Fig. 4. This type of signature was first reported by Guo and Krider^40^. The dart leader luminosity, at 777 nm, lasts on average for 0.24±0.18 ms and has an amplitude which is a fraction of 19.5 ± 6.7% of the return stroke peak luminosity. For these 33 dart leaders, the luminosity has sufficient signal-to-noise to allow for a temperature estimate. The average temperature among the 33 dart leaders is 15.1 ± 3.6 kK, which is lower than the $\sim$22 kK figure reported by Chang et al.^24^. The dart leaders have comparable temperatures to the return strokes they create. The temperature difference is normally distributed with average and standard deviation of 3.5 ± 5.2 kK, meaning that the return strokes are only 3.5 kK hotter than the preceding dart leaders (on average), but sometimes the dart leaders can be hotter. The top row of Fig. 4 shows two example dart leaders within the same flash that were imaged with the Chronos camera (*t* = −18 and −0.7 ms), as well as the return stroke created by the second one (shown by selecting a post-saturation frame at *t* = 2 ms). The rest of Fig. 4 reports on the dart leader-return stroke sequence happening at *t* = 0. This recording is very representative of our data set. The strike took place 4.7 km away. The average temperature of the dart leader and the ensuing return stroke are 17.4 kK and 18.8 kK, respectively. It is remarkable that, despite the intense optical surge caused by channel re-ignition during the RS, the two temperatures differ only slightly.

Figures 1b and 4e are quite representative of the difference between optical signatures of RSs that have new ground contacts versus the ones that use previously-established channels. New ground contacts have a sharper rise from zero luminosity leading to a larger peak. Meanwhile, RSs using previously-existing channels exhibit a slow luminosity ramp (leading to up to 20% of the ensuing RS stroke peak luminosity), which is associated with the precursor dart leader. These features are consistent with previous findings by Quick and Krider^31^, and they have the potential to serve as an automated tool to detect new ground contact points, something that is quite challenging to do solely with radio data from long-range lightning detection networks^41^.

#### M-component and continuing current

Figure 5 depicts a flash 4.2 km away with several M-components, which become apparent to SOPAPILLA only during the peak luminance of each one of them. The M-components can be seen in the figure as bumps in electric field change (5d) and photodiode luminosity (5e) signatures. This flash contained 4 return strokes reported by ENTLN and there was 55 ms of continuing current after the first return stroke according to the slow antenna data (not shown). Additionally, there was continuing current after each consecutive return stroke for a total of 300 ms of continuing current throughout the entire flash. Figure 5 only depicts the first return stroke as this was the only one with bright enough M-components to be seen with the chosen gain setting. In addition to the photodiode light curves, Fig. 5e shows the integrated intensity from the Chronos camera scaled to the photodiode range to demonstrate the persistence of signal (up to at least 20 ms) despite it dropping in and out of the photodiode sensitivity. Figure 5e has been split into 5e$_1$ (21-ms record) and 5e$_2$ (first 2.4 ms) to aid with visualizing the Chronos integrated intensity.

The M-components increase the channel luminosity to a level which allows for a temperature estimate. The average temperature of the M-components is 23.5 kK, but the discrepancy between the two ratios is large. In Fig. 5e, the light curve obtained from integrating the luminosity in each Chronos frame indicates that the channel maintains a minimum brightness level for at least 20 ms. There are five periods in which the M-component temperature can be estimated, with the last one ending at 10 ms. If we fit the temperature trend we find that: (i) the temperatures of the M-components are lower than the temperatures in the preceding RS, and (ii) the channel seems to cool down over time at a rate of about 1.5 kK/ms. We interpret these findings as evidence that the RS channel with continuing current maintains a high temperature of >20 kK for at least 10 ms. The M-component temperatures and rates of temperature decay reported here are comparable to values reported by Wang et al.^26^, who had figures of the order of $\sim$16 kK (from their Fig. 7a–d) and $\sim$3 kK/ms (from their Fig. 7c only). The primary conclusion here is that a return stroke with continuing current cools down slowly. The cooling rates reported above are two orders of magnitude slower than the ones reported by Boggs et al.^11^. These authors report cooling rates of $\sim$100 kK/ms within the first millisecond of the lifetime of strokes with no reported continuing current.

## Summary

Previous instruments and methods for finding the temperature of lightning have only yielded a small amount of data. The proposed instrument, SOPAPILLA, significantly increased the available information on the temperatures of return strokes and dart leaders during a single deployment in the Summer of 2024. This study improves on the photometric temperature method by adding both measurements of the continuum radiation and context videos. These advancements produced measurable temperatures more frequently and with smaller error bars than the results previously published, which relied on an estimate of the continuum radiation intensity^7^. We reported the peak temperature of 23 return strokes and showed that they increase weakly with peak current ($I_p$), as $\propto$ $I_p^{0.21}$. We report the temperature of a powerful stepped leader channel at about 25 kK, with step-wise local increases in temperature around large steps. Across 33 dart leader-return stroke sequences, we found that the dart leader has a comparable temperature (15 kK) to the ensuing return stroke, which was only 3.5 kK hotter. By measuring the temperature of the brightest intervals of four M-components we determined that the return stroke channel with continuing current can sustain a temperature of 20 kK for at least 10 ms, cooling at a rate of 1.5 kK/ms. In the future, we plan to (i) continue deploying SOPAPILLA to increase available data on the temperature of lightning sub-processes, and (ii) use this data to constrain the energetic and chemical impacts of all types of sub-processes.

## Methods

### Instruments

**Fig. 6:**
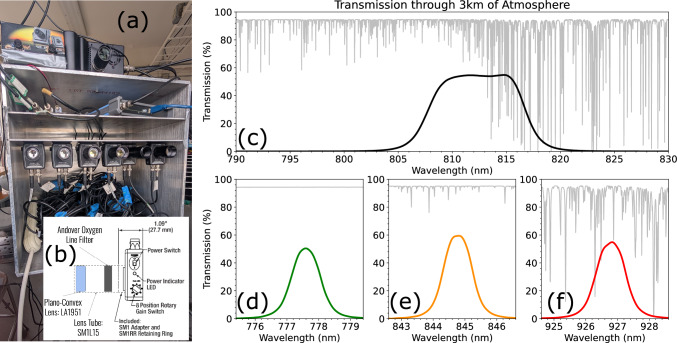
**a** A picture of SOPAPILLA deployed at Langmuir Lab, the top of the box has a mount for the Chronos camera, the top shelf contains the PicoScope and electric field antennae circuit, the bottom shelf has all 6 photodiodes mounted. **b** A diagram of each photodiode from the Thorlabs manual^45^ augmented with lens and filter used. **c**–**f** Near-infrared radiation transmission through 3 km of atmosphere (gray trace) and the transmission of each filter placed in front of the photodiodes (colored, Gaussian-shaped traces).

SOPAPILLA is a 6-channel photometer array shown in Fig. 6a. In this article, we focus the analysis on the data collected with four Thorlabs PDA100A2 photodiodes. Each photodiode was set up so to have an approximate FOV spanning 20$^\circ$, as determined by the optics shown in Fig. 6b. The gain was set to 20 dB, resulting in a 1.25 $\upmu$s rise time, as reported by ThorLabs. The continuum filter is an approximately square filter centered on 812 nm with a width of about 10 nm, as shown in Fig. 6c. This spectral region was selected because it has no oxygen or nitrogen transition lines. The three oxygen line filters have been described previously^7^, but were near-gaussian with central wavelengths at: 777.59, 844.77, and 926.78 nm (Fig. 6d–f). All filters have an index of refraction of 2.05 and their out of band blocking averages $10^{-4}$ from X-ray to far-infrared. SOPAPILLA includes a Chronos context camera recording at 1000 fps with an approximate FOV similar to the photodiodes.

Much like in the Summer of 2023^7^, we deployed this optical setup in 2024 alongside a fast and slow electric field change antenna and connected all instruments to a PicoScope 4824A and digitized the data at 800 kHz. However, to trigger both the Chronos camera and the GPS timing unit, we connected them in series to the trigger output on the PicoScope. These data sets were analyzed in tandem with Earth Networks Total Lightning Detection (ENTLN) data, which yielded location and peak current.

### Data analysis

Using this instrument, we replicate the slitless lightning spectroscopic method for determining the temperature, which involves the ratio between two emission line intensities from the same species. However, filtered photodiode measurements differ from traditional spectroscopic measurements in a couple of specific ways.

First, the filters placed in front of the photodiodes are Fabry–Perot filters. This means that when incoming light enters the filter at a non-zero angle of incidence the central wavelength of the filter’s bandwidth shifts towards higher frequencies (a blueshift). When applied to our observations of emission lines, the filters eventually blueshift enough that they begin to miss the emission line they are measuring. So as the angle of incidence increases, the effective transmission of the emission line, $\mathcal {T}_\textrm{j}(\lambda )$, decreases for any *j*-th filter. However, we mitigate this in 2 separate ways: the filters are all slightly redshifted compared to their emission lines and the filters are all placed behind a focusing lens which halves the effect of the blueshift^42^. In total, when each filter is put in ratio with another the blueshift does not significantly affect measurements within the central $6^\circ$ of the FOV. For the 926/777 ratio the error is never more than 21%, which is lower than the error bars we report for the majority of our error weighted temperatures. For the 926/844 ratio, the error is below 30% for about half of our FOV. Since the effect of the blueshift on our temperature measurements is small and the randomness innate to the path of lightning would make choosing a single angle of incidence difficult, we have chosen to neglect the blueshift entirely. This means that $\mathcal {T}_{j}(\lambda )$ is only a convolution of the atmospheric transmission, the *j*-th filter’s transmission at $0^\circ$ incidence, and the responsivity of the photodiode. The first two are given in Fig. 6d–f, while the third one was previously given in Fig. 1 of Wemhoner et al.^7^.

Second, photodiodes differ from traditional spectroscopic measurements because they yield a summation of both the continuum radiation and the line intensity over the bandwidth of the filter. To retrieve just the integrated line intensity from a photodiode, we take the simultaneous continuum measurement and convert it to the correct wavelength and spectral width to simulate a continuum measurement for each line. This is done by assuming the continuum is described by a blackbody at 7 kK, as suggested by Kieu et al.^43^. Then, we integrate over this blackbody continuum multiplied by the normalized transmission of the filter and divide by the width of the filter as shown in Eq. (1).

1 $$\begin{aligned} C_{j} = \frac{\int \mathcal {T}_{j}(\lambda )\cdot B_{\lambda }(7 \, \textrm{kK})d\lambda }{D_j} \, \text {, where, } D_j = \int \mathcal {T}_{j}(\lambda )d\lambda \, , \end{aligned}$$

and $B_{\lambda }(7\text { kK})$ is the Blackbody spectral radiance at 7 kK. Next, we can translate the continuum measurement to each filter by taking a ratio of the line filter to the continuum filter. This allows us to calculate the line intensity for each narrowband filter given the measurements, the line normalization factor (*j* = line = 777, 844, or 926), and the continuum normalization factor (*j* = cont) as shown in Eq. (2) below:

2 $$\begin{aligned} I_\textrm{line} = \frac{M_\textrm{line}}{D_\textrm{line}} - \frac{C_\textrm{line}}{C_\textrm{cont}} \frac{M_\textrm{cont}}{D_\textrm{cont}} \, , \end{aligned}$$

where $I_\textrm{line}$ is the integrated intensity of the oxygen line within one of the three filters and *M* is the measurement from the photometers after a relative calibration to account for the manufacturing differences in sensitivity. Note that the ratios of normalization factors in Eq. (2) cancels out the units of the Blackbody spectral radiance. We can then plot the traditional spectroscopic equation^9^, reproduced in Eq. (3), over a range of temperatures and numerically invert this to get a function that accepts a ratio and returns a temperature for each viable ratio as previously demonstrated^7^. In local thermodynamic equilibrium conditions, the ratio between the intensity of two transition lines of the same species can be calculated as:

3 $$\begin{aligned} \frac{I_{926}}{I_{777}} = \frac{\sum _{n=1}^9 g_{n}A_{n}\nu _{n}e^{-\frac{\epsilon _{n}}{\kappa _bT}}}{\sum _{m=1}^3 g_{m}A_{m}\nu _{m}e^{-\frac{\epsilon _{m}}{\kappa _bT}}} \equiv f(T_{926/777}) \, , \end{aligned}$$

where the *n* and *m* summations account for all possible oxygen transition lines around 926 nm and 777 nm, respectively. For each line, *g* is the statistical weight of the transition, *A* is the Einstein coefficient, $\nu$ is the frequency of the emitted photon (given from the wavelength as $c/\lambda$), and $\epsilon$ is the energy of the excited state. Additionally, $\kappa _b$ is the Boltzmann constant and *T* is the temperature of the plasma. This makes the intensity ratio purely a function of temperature, *f*(*T*), which allows us to numerically invert the function so that the ratio of the intensity measurements yield a temperature when plugged into $f^{-1}$. All atomic transition constants used in equation (3) are sourced from the National Institute of Standards and Technology (NIST)^44^. Repeating this process for the other ratio, $I_{926}/I_{844}$, yields a different temperature measurement and both of these measurements yield upper and lower bounds for the temperature. This allows us to report an error-weighted temperature measurement. To do this, for each ratio we test what the temperature would be if the ratio was 5% lower ($T_\textrm{low}$) or 5% higher ($T_\textrm{high}$). This simulates an error in the measurement and the difference between these two simulated temperatures gives an approximation of the mathematical error at that originally measured temperature. Using this mathematical error we perform an error-weighted average of the two temperatures, given as:

4 $$\begin{aligned} T_\textrm{reported} = \frac{E_{926/844}T_{926/777}+E_{926/777}T_{926/844}}{E_{926/777}+E_{926/844}}\, , \end{aligned}$$

where the error is defined as *E* = $T_\textrm{high} - T_\textrm{low}$ for each ratio, and $T_\textrm{reported}$ is the error-weighted average temperature reported in all of our figures, which corresponds to the FOV averaged, atomic oxygen photometric temperature. Since this is averaged over the FOV and for atomic oxygen only, this quantity is only a proxy for the real temperature. As discussed previously^7^, this temperature tends to be lower than what is reported through spectroscopy due to two main reasons: (i) the averaging over the FOV, and (ii) the hottest channel portion near the ground is often obscured by a mountain in the FOV.

## Data Availability

Data used in this study has been made publicly available online: https://doi.org/10.5281/zenodo.16883559. The Earth Networks Total Lightning Detection Network (ENTLN) data is freely available upon request from AEM Earth Networks. ENTLN data requests can be made at: https://www.earthnetworks.com/product/lightning-data/.
